# Supplementary material for: Transcriptome Analysis Reveals Vimentin-Induced Disruption of Cell–Cell Associations Augments Breast Cancer Cell Migration
Source: Cells. 2022 Dec 13;11(24):4035. doi: 10.3390/cells11244035 (PMC9776984; doi:10.3390/cells11244035)
Supplement: Supplementary file 1 [file cells-11-04035-s001.zip › DEGs and enrichment analysis.zip!/DEGs and enrichment analysis/WT_VIMvsVector KEGG/ALL/WT_VIMvsVector_ALL.pathway_map.zip!/WT_VIMvsVector_ALL.pathway_map/src/hsa05166.html]

hsa05166


- hsa:6513

- Up regulated genes

ENSG00000117394(1.94254957435037)

- hsa:7042

- Down regulated genes

ENSG00000092969(-2.32734226266555)

- hsa:107

- Up regulated genes

ENSG00000164742(1.87205666251591)

- hsa:7042

- Down regulated genes

ENSG00000092969(-2.32734226266555)

- hsa:991

- Up regulated genes

ENSG00000117399(2.41242866521263)

- hsa:9133

- Up regulated genes

ENSG00000157456(1.60409075038332)

- hsa:7015

- Up regulated genes

ENSG00000164362(2.48262040043416)

- hsa:1870

- Up regulated genes

novel.507(3.55620908288126)
- hsa:1869

- Up regulated genes

ENSG00000101412(1.977544660594)

Close
